# C-terminal domain small phosphatase 1 (CTDSP1) regulates growth factor expression and axonal regeneration in peripheral nerve tissue

**DOI:** 10.1038/s41598-021-92822-8

**Published:** 2021-07-14

**Authors:** Noreen M. Gervasi, Alexander Dimtchev, Desraj M. Clark, Marvin Dingle, Alexander V. Pisarchik, Leon J. Nesti

**Affiliations:** 1 Laboratory of Clinical and Experimental Orthopaedics, Department of Surgery, Uniformed Services University of Health Sciences, 4801 Rockville Pike, Bethesda, MD 20889 USA; 2 Henry M. Jackson Foundation for the Advancement of Military Medicine, 6720A Rockledge Drive, Bethesda, MD 20817 USA; 3 Alcamena Stem Cell Therapeutics, 1450 South Rolling Road, Suite 4.069, Halethorpe, MD 21227 USA; 4 Department of Orthopaedics and Rehabilitation, Walter Reed National Military Medical Center, 8901 Rockville Pike, Bethesda, MD 20889 USA

**Keywords:** Neuroscience, Epigenetics in the nervous system, Peripheral nervous system, Regeneration and repair in the nervous system, Translational research

## Abstract

Peripheral Nerve Injury (PNI) represents a major clinical and economic burden. Despite the ability of peripheral neurons to regenerate their axons after an injury, patients are often left with motor and/or sensory disability and may develop chronic pain. Successful regeneration and target organ reinnervation require comprehensive transcriptional changes in both injured neurons and support cells located at the site of injury. The expression of most of the genes required for axon growth and guidance and for synapsis formation is repressed by a single master transcriptional regulator, the Repressor Element 1 Silencing Transcription factor (REST). Sustained increase of REST levels after injury inhibits axon regeneration and leads to chronic pain. As targeting of transcription factors is challenging, we tested whether modulation of REST activity could be achieved through knockdown of carboxy-terminal domain small phosphatase 1 (CTDSP1), the enzyme that stabilizes REST by preventing its targeting to the proteasome. To test whether knockdown of CTDSP1 promotes neurotrophic factor expression in both support cells located at the site of injury and in peripheral neurons, we transfected mesenchymal progenitor cells (MPCs), a type of support cells that are present at high concentrations at the site of injury, and dorsal root ganglion (DRG) neurons with *REST* or *CTDSP1* specific siRNA. We quantified neurotrophic factor expression by RT-qPCR and Western blot, and brain-derived neurotrophic factor (BDNF) release in the cell culture medium by ELISA, and we measured neurite outgrowth of DRG neurons in culture. Our results show that CTDSP1 knockdown promotes neurotrophic factor expression in both DRG neurons and the support cells MPCs, and promotes DRG neuron regeneration. Therapeutics targeting CTDSP1 activity may, therefore, represent a novel epigenetic strategy to promote peripheral nerve regeneration after PNI by promoting the regenerative program repressed by injury-induced increased levels of REST in both neurons and support cells.

## Introduction

Peripheral nerve injury (PNI) constitutes a major clinical and economic burden. Despite the regenerative ability of peripheral nerves, the slow rate of regeneration and the limited time frame during which end-organ reinnervation has to occur to be successful mean that complete functional recovery is rarely achieved, even after surgical treatment, for injuries that leave a gap between nerve stumps more than a few centimeters wide or proximal injuries located more than 30 cm from the target organ. This is because axons either fail to reach their target organ or the target organ loses its ability to be re-innervated, a phenomenon that seems to happen between 12 and 18 months after injury. As a consequence, many patients are left with motor and/or sensory disability and often develop chronic pain, all of which can greatly affect a patient’s quality of life.

**Figure 1:**
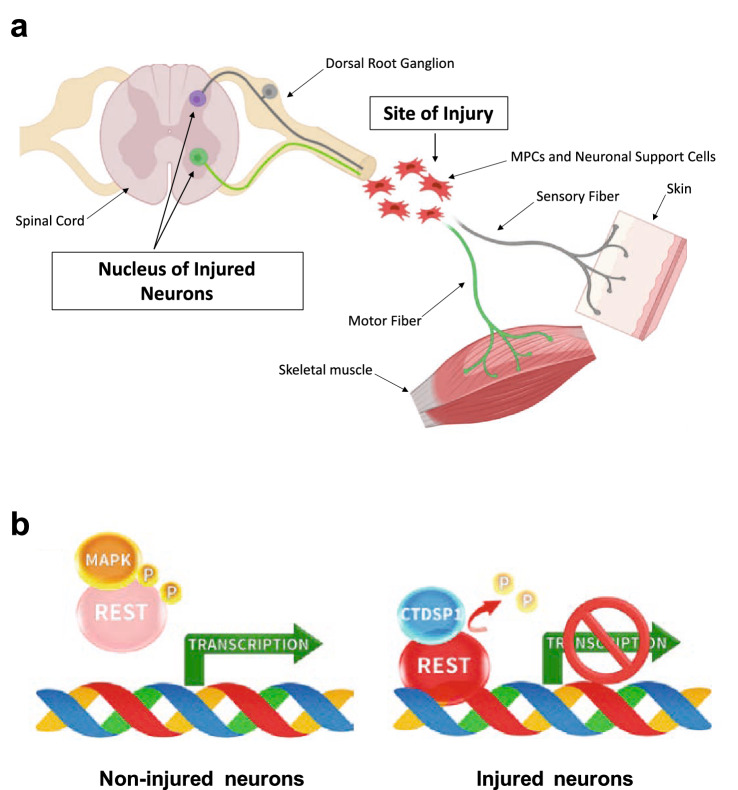
Injury model and REST-CTDSP1 regulation of neuronal gene transcription. (**a**) Schematic illustrating our working hypothesis. After musculoskeletal injury, MPCs and other support cells (shown in red) accumulate at the site of injury. We propose to promote axonal regeneration and support axonal growth by modulating REST levels both in the nucleus of supporting cells at the site of injury and in the nucleus of injured neurons. (**b)** Left panel: In non-injured neurons, phosphorylation of REST by a MAP kinase (MAPK) leads to detachment of REST from the chromatin and expression of neuron-specific genes. Right panel: After injury, desphosphorylation of REST by the C-terminal domain small phosphatase 1 (CTDSP1) protects REST from degradation and allows REST-mediated repression of neuron-specific genes.

Regeneration of peripheral nerves relies on the ability of peripheral neurons to switch to a regenerative state and on the development of a pro-regenerative environment at the site of injury created by local support cells. Both events require comprehensive transcriptional changes in both injured neurons and support cells^1–4^; however, therapeutic approaches have mainly focused on modulating or introducing a single or at most the combination of a limited number of molecules, rather than sustaining the regenerative program as a whole^5–8^. An overall epigenetic reprogramming approach could promote a favorable regenerative environment by targeting the support cells located at the site of injury, as well as activating the intrinsic mechanism of axonal regeneration in the neuronal cell bodies of the injured nerves located at a distance from the site of injury (Fig. 1a). While research has mostly focused on administering pro-regenerative factors, little attention has been given to releasing the break on pro-regenerative genes. Importantly, the expression of many genes required for neuronal survival, axonal growth, synaptic plasticity, vesicular transport and ionic conductance is repressed by a single master transcriptional repressor known as Repressor Element-1 (RE-1) Silencing Transcription Factor (REST)^9–11^. REST levels and activity are tightly controlled through both transcriptional and post-transcriptional mechanisms. At the protein level, mitogen-activated protein kinase (MAPK)-mediated phosphorylation of serines 861 and 864 targets REST for proteasomal degradation through interaction with beta-transducin repeat containing E3 ubiquitin protein ligase (βTrCP)^12^. Conversely, dephosphorylation of the same serines by CTDSP1, which is recruited by REST to the REST complex, stabilizes REST and allows repression of genes downstream of the RE-1 element (Fig. 1b)^12^.

While REST levels are kept low in healthy mature neurons through active degradation to maintain the chromatin clear of this gene repressor and allow expression of neuronal genes, both in vitro and in vivo studies have shown that REST levels are increased after injury^13–16^. This increase in REST immediately after injury is thought to allow neurons to enter a regenerative state^13^. However, a sustained upregulation of REST after trauma is responsible for inhibition of regeneration and development of PNI-associated chronic pain^14^.

Among support cells, Schwann cells in the distal stump play a fundamental role in supporting the regenerative process by providing guidance for the regenerating axon and promoting neuronal survival through secretion of neurotrophic factors. After injury, Schwann cells undergo a phenotypic change that is triggered by losing contact with the axon, and convert to what is known as “repair Schwann cell”^17,18^. However, these denervated Schwann cells lose their ability to promote regeneration within 1 to 3 months after injury^19^, a timeframe that is too short for many injuries given the slow speed of nerve regeneration. In addition to losing their ability to sustain regeneration, chronically denervated Schwann cells also have an inhibitory effect on nerve regeneration^20^. In an attempt to overcome this short time frame issue, some studies have investigated the use of adult mesenchymal stem cells (MSCs) as Schwann cells extenders to promote regeneration. Introduction of exogenous stem cells has demonstrated promising results, but these studies are limited in that they have focused on obtaining cells from remote locations in the body—a strategy that can be both technically challenging and complicated from a FDA regulatory standpoint^21^. Thus, alternative autologous sources of cells would be clinically useful. We have previously reported a robust endogenous population of mesenchymal progenitor cells (MPCs) which are present at high concentrations at the site of neuromusculoskeletal injury and exhibit trophic and pro-regeneration properties that are similar to that of bone marrow derived MSCs^22,23^. These include modulation of inflammatory responses and secretion of neurotrophic factors, including brain-derived growth factor (BNDF), nerve growth factor (NGF), ciliary neurotrophic factor (CNTF) and neurotrophin 3 (NT-3)^22^, all of which have specific functions in peripheral nerve regeneration to promote the growth of axons and migration of Schwann cells into the site of injury. The expression of these factors can be enhanced by culturing MPCs in a defined medium for neurotrophic induction^23^. Importantly, we have shown that culturing dorsal root ganglia (DRGs) with MPCs or in MPC conditioned medium results in an increase in the density and length of neurites that extend from DRGs^23^. Given their abundant presence at the site of injury and their general pro-regenerative functions, MPCs represent an attractive target to enhance the regeneration processes that promote nerve repair at the site of injury. However, it is not known if the CTDSP1-REST pathway is functional in MPCs and whether CTDSP1 knockdown promotes MPC expression of neurotrophic factors.

Modulation of REST levels after injury could at the same time sustain the neuronal intrinsic regeneration program, promote synapsis formation at the end-organ, prevent development of chronic pain and promote neurotrophic factor release from support cells to create a favorable environment at the site of injury. So far, direct regulation of transcription factors has resulted difficult to achieve because of the need to target protein–protein or protein-DNA interactions. An alternative strategy may therefore be to modulate REST activity indirectly by targeting CTDSP1, the phosphatase that protects it from degradation. While CTDSP1 knockdown has been shown to promote neurotrophic factors expression in HEK-293 cells^24^, it is not known whether it effectively promotes neurotrophic factor expression in neurons or in support cells. Here, we investigate whether the REST pathway is active in MPCs. We then test the hypothesis that inhibition of CTDSP1 function promotes neurotrophic factor expression in both MPCs and neurons and stimulates neurite regeneration.

## Results

### CTDSP1 knockdown promotes neurotrophin release from primary human MPCs

**Figure 2:**
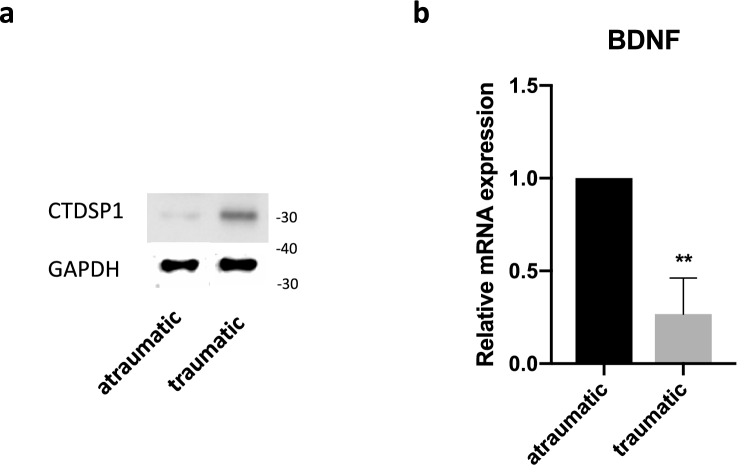
Traumatic injury results in increased CTDSP1 and decreased BDNF expression. (**a**) Western blot of CTDSP1 protein in muscle tissue. Full-length blot is shown in Supplementary Figure S1. (**b**) Quantification of mRNA expression in traumatized muscle tissue by qRT-PCR. **p < 0.01, two-tailed Student’s *t*-test with n = 3 biological replicates, error bars are SD.

**Figure 3:**
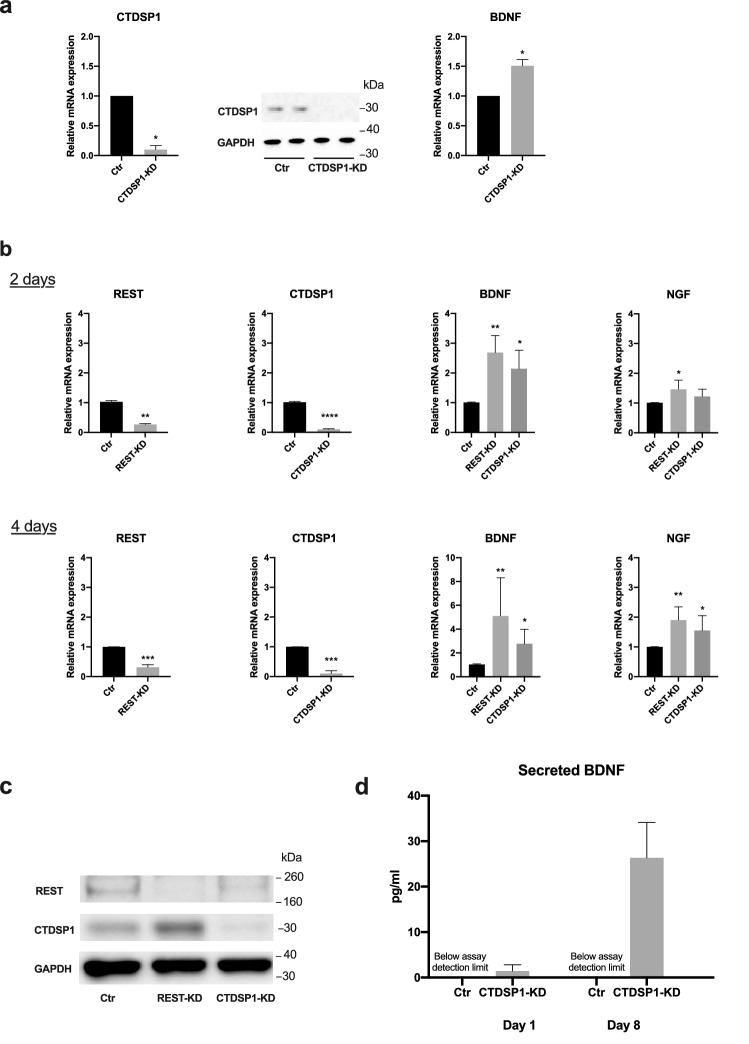
Knockdown of REST or CTDSP1 increases BDNF expression. (**a**) Quantification of mRNA and protein expression in HEK-293 cells 48 h after transfection of CTDSP1 siRNA. Knockdown of CTDSP1 was confirmed by qRT-PCR and Western blot analysis. BDNF mRNA levels were quantified by qRT-PCR. *p < 0.05, paired Student’s *t*-test with n = 3 biological replicates, error bars are SD. (**b**) mRNA expression levels in cultured MPCs 2 days and 4 days after transfection of siRNA. *p < 0.05, **p < 0.01, ***p < 0.0001, ****p < 0.00001, two-tailed paired Student’s *t*-test (REST and CTDSP1) or Kruskal–Wallis test with Dunn’s multiple comparisons test (BDNF and NGF), with n = 5 biological replicates, error bars are SD. (**c**) Western blot analysis of protein expression levels of CTDSP1 and REST in MPCs 4 days after transfection of REST or CTDSP1 siRNA. GAPDH was used as loading control. Protein quantification shows a 75% reduction in REST protein after CTDSP1 knockdown. (**d**) Concentration of BDNF secreted in the MPC supernatant over 24 h measured by ELISA 1 day and 8 days after transfection of CTDSP1 siRNA. Control samples showed signal below the detection limit of the assay. Four samples were analyzed for each condition, error bars are SD. For all graphs: Ctr = control scramble siRNA, KD = knockdown. Full-length blots are shown in Supplementary Figures S2 and S3.

Peripheral nerves are often injured as a result of musculoskeletal trauma. To determine how traumatic injury affects CTDSP1 protein expression at the site of injury, we ran Western blot analysis on total protein lysate from human traumatized and non-traumatized muscle tissue. Our results showed a tenfold increase in CTDSP1 protein after traumatic injury (Fig. 2a). Because of the role of CTDSP1 in stabilizing REST, we reasoned that an increase in CTDSP1 protein would be associated to a decreased transcription of neurotrophic factors. To test this hypothesis, we quantified BDNF mRNA by RT-qPCR. Results showed about 75% decrease in BDNF mRNA in traumatized muscle tissue compared to non-traumatized tissue (0.2667 ± 0.1948 relative to control, *p* = 0.0029) (Fig. 2b). Based on these results, we tested whether downregulation of CTDSP1 promotes neurotrophic factor transcription. To test the efficiency of the CTDSP1-specific siRNA and the effect of CTDSP1 knockdown on neurotrophin expression, we initially transfected HEK-293 cells with *CTDSP1*-specific siRNA. A scramble siRNA not targeting any known mammalian sequence was used as negative control. Cells were lysed two days after transfection and knockdown of CTDSP1 mRNA and protein was confirmed by RT-qPCR and Western blot (Fig. 3a). Results showed a 90% knockdown of CTDSP1 mRNA (0.1015 ± 0.06551 expression relative to control, *p* = 0.0328) and 97% knockdown of CTDSP1 protein (CTDSP1 siRNA: 0.027 ± 0.008 relative to control, *p* = 0.0003). Quantification of BDNF mRNA by RT-qPCR showed increased levels of *BDNF* after CTDSP1 knockdown (1.507 ± 0.1046 expression relative to control, *p* = 0.0139) (Fig. 3a), indicating that CTDSP1 regulates BDNF expression.

Next, we investigated whether modulation of the REST pathway promotes neurotrophic factor expression in neuronal support cells. Specifically, we employed primary human MPCs because these cells are abundant at the site of injury and represent a clinically relevant source of autologous cells. To test our hypothesis that MPC expression of neurotrophic factors can be potentiated by modulating the CTDSP1-REST pathway, we transfected cultured MPCs from five independent donors with *REST-* or *CTDSP1*-specific siRNAs. RNA expression analysis showed increased levels of *BDNF* and *NGF* after either REST or CTDSP1 knockdown, both two days (REST knockdown: REST 0.2625 ± 0.03352 *p* = 0.0012, BDNF 2.685 ± 0.5747 *p* = 0.0014, NGF 1.462 ± 0.3056 *p* = 0.0138; CTDSP1 knockdown: CTDSP1 0.1002 ± 0.02074 *p* < 0.0001, BDNF 2.144  ± 0.6211 *p* = 0.0353, NGF 1.223 ± 0.2403 *p* = 0.3352) and four days (REST knockdown: 0.3158 ± 0.08239 *p* = 0.0005, BDNF 5.108 ± 3.202 *p* = 0.0025, NGF 1.901 ± 0.4445 *p* = 0.0018; CTDSP1 knockdown: 0.1009 ± 0.09295 *p* = 0.0003, BDNF 2.768 ± 1.204 *p* =  0.0402, NGF 1.550 ± 0.5028 *p* = 0.0403) after transfection (Fig. 3b). To verify that knockdown of CTDSP1 induces a decrease in REST protein expression, we ran Western blot analysis on total protein lysate from MPCs four days after transfection with either *REST-* or *CTDSP1*-specific siRNA. Our result showed a 96% decrease in REST protein after REST knockdown and a 75% decrease after CTDSP1 knockdown (Fig. 3c). This result supports our hypothesis that CTDSP1 modulates neurotrophin levels through stabilization of REST. In order to support axonal survival and growth at the site of injury, BDNF needs to be secreted from the MPCs and support cells surrounding the injured nerves. To test whether an increase in BDNF mRNA after CTDSP1 knockdown translates into an increase in secreted BDNF, we quantified BDNF protein levels in cell culture supernatants by ELISA. While secreted BDNF was below the detection limits in the supernatants of MPCs transfected with control siRNA both one day and eight days after transfection, the supernatant of cells transfected with CTDSP1-specific siRNA contained detectable levels of BDNF as soon as one day after transfection (day 1: 1.423 ± 1.414 pg/ml; day 8: 26.33 ± 7.772 pg/ml) (Fig. 3d). Taken together, these results demonstrate that CTDSP1 KD in human MPCs at the site of injury enhances neurotrophic factor production.

### CTDSP1 knockdown promotes BDNF expression and neurite outgrowth in primary DRG neurons

**Figure 4:**
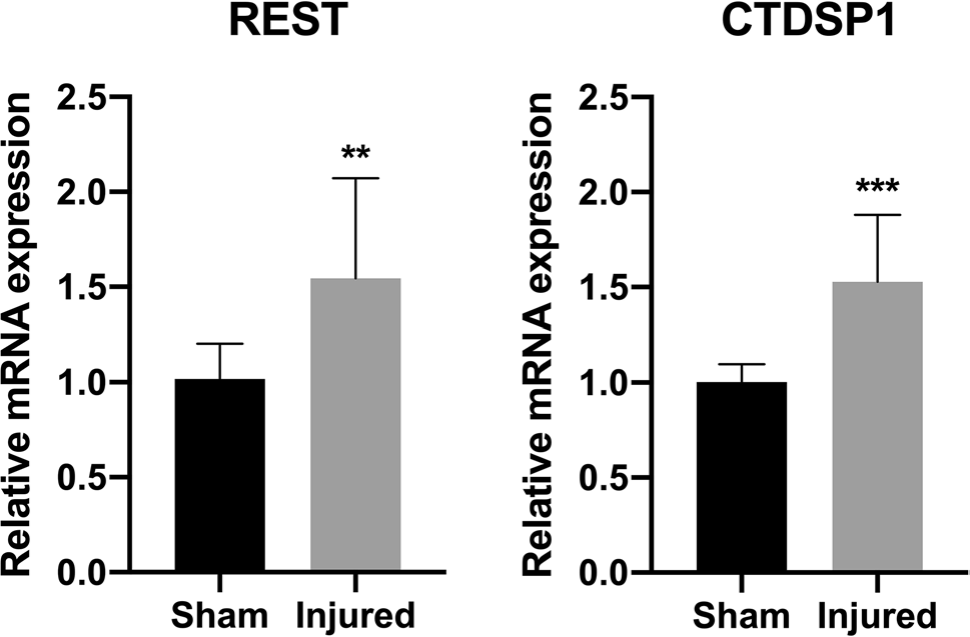
REST and CTDSP1 are upregulated after peripheral nerve injury. qRT-PCR analysis of mRNA expression levels in L3-L5 DRGs dissected 24 h after sciatic nerve injury or sham surgery. **p < 0.01, ***p < 0.001, Mann–Whitney test, n = 10 DRGs from three rats for sham, n = 7 DRGs from three rats for injured, error bars are SDs.

**Figure 5:**
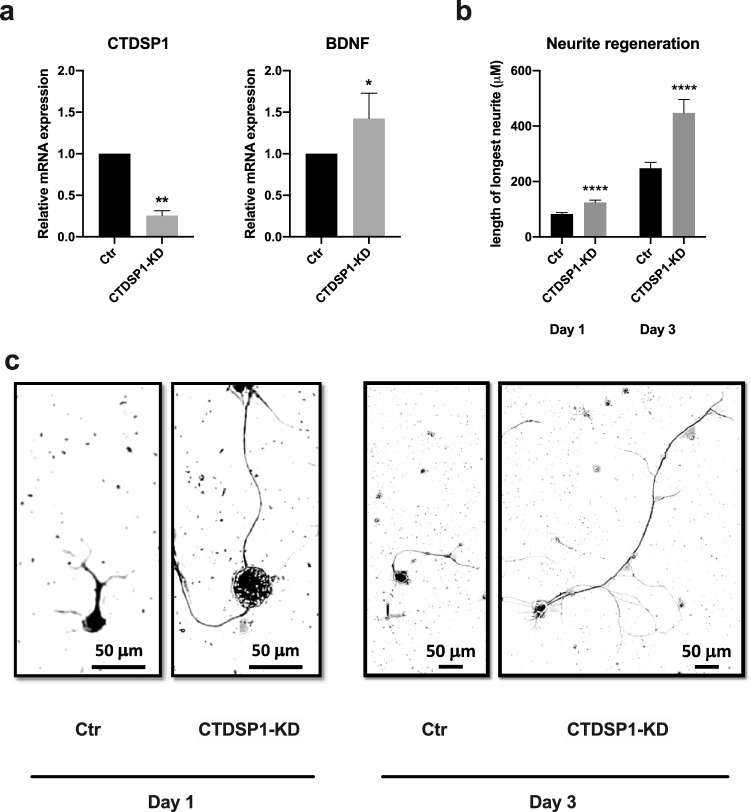
Knockdown of CTDSP1 in DRG neurons promotes BDNF expression and neurite regeneration. (**a**) qRT-PCR analysis of mRNA expression levels in cultured DRG neurons 24 h after CTDSP1 siRNA transfection. * *p* < 0.05, ** *p* < 0.01, paired two-tailed Student’s *t*-test with n = 4 biological replicates, error bars are SD. (**b**) Quantification of the length of the longest neurite of dissociated DRG neurons in culture 1 day and 3 days after REST or CTDSP1 knockdown. ****p < 0.0001, Mann–Whitney test, n = min 68 neurites from 3 biological replicates, error bars are SEM. (**c**) Representative images of neurons after CTDSP1 knockdown. Ctr = scramble siRNA control, KD: knockdown.

In addition to supporting axonal survival and growth at the site of injury, we propose to promote neuronal survival and axonal regeneration by acting directly on the neuronal cell bodies, which are often located several centimeters from the site of injury. REST has been shown to increase in DRG neurons after PNI^14,15^. To confirm that REST increases and test how CTDSP1 levels vary after PNI, we performed sciatic nerve transection or control sham surgery on adult rats. One day after surgery, rats were sacrificed and the DRGs containing the neurons of the sciatic nerve were collected. qRT-PCR analysis showed that both *REST* and *CTDSP1* mRNA increased significantly in the neurons of injured sciatic nerve compared to neurons of sciatic nerve of rats that received sham surgery (REST injured: 1.5460 ± 0.5260 relative to sham, *p* = 0.0068; CTDSP1 injured : 1.528 ± 0.325 relative to sham, *p* = 0.0002) (Fig. 4). To test our hypothesis that modulation of neuronal REST levels through CTDSP1 promotes neurite outgrowth through an increase in neurotrophic factor production, we transfected cultured primary DRG neurons from adult rats with CTDSP1 siRNA. RT-qPCR analysis of CTDSP1 and BDNF mRNA expression one day after transfection, showed a 75% decrease in CTDSP1 mRNA (0.2566 ± 0.05787 relative to control, *p* = 0.0084) and a 40% increase in BDNF mRNA (1.425 ± 0.3030 relative to control, *p* = 0.0127) after CTDSP1 knockdown (Fig. 5a). These results indicate that CTDSP1 knockdown increases neuronal expression of BDNF. To test whether CTDSP1 knockdown promotes regeneration, we cultured dissociated primary DRG neurons from adult rats and measured the length of the longest neurite 1 day and 3 days after transfection of REST or CTDSP1 siRNA^25^. As early as one day after transfection, neurites of neurons transfected with CTDSP1 siRNA were significantly longer than those growing from neurons transfected with a control siRNA (mean length: control 82.91 ± 4.872 µm; *p* < 0.0001; CTDSP1 knockdown: 124.7 ± 8.243 µm, *p* < 0.0001) (Fig. 5b,c). Similarly, increased neurite length was observed after CTDSP1 knockdown 3 days after transfection (mean length: control 247.7 ± 21.75 µm; CTDSP1 knockdown 447.4 ± 48.50 µm, *p* < 0.0001) (Fig. 5b,c). Taken together, these results show that knockdown of CTDSP1 in primary neurons increases the expression of the neurotrophic factor BDNF and leads to increased neurite regeneration.

## Discussion

In this study, we provide evidence that the REST-CTDSP1 pathway is active in MPCs and that modulation of REST and/or CTDSP1 increases neurotrophic factor expression in both MPCs and DRG neurons, and promotes neurite regeneration in DRG neurons.

First, we show that knockdown of either REST or CTDSP1 in MPCs results in increased levels of BDNF and NGF mRNA. We support this finding by showing that CTDSP1 knockdown leads to increased levels of BDNF protein in cell culture medium. Second, we show that knockdown of CTDSP1 in DRG neurons increases expression of BDNF mRNA. Finally, we show that CTDSP1 knockdown promotes neurite regeneration by comparing the length of DRG neurites between CTDSP1 and control siRNA transfected neurons.

Our results are in accordance with previous studies which have reported an increase in BDNF expression after CTDSP1 knockdown in HEK-293 cells^24^. However, this is the first time, to our knowledge, that CTDSP1 knockdown has been shown to increase neurotrophic factor expression in MPCs and adult DRG neurons. Importantly, our study demonstrates that the REST pathway is active in MPCs. MPCs are abundant at the site of traumatic injury and may therefore be a clinically useful source of autologous cells, circumventing the issues associated with the scarcity of Schwann cells or mobilization of MSCs from the bone marrow^26,27^. We have previously shown that MPCs possess neurotrophic properties equivalent to those of MSCs, and express BDNF at significantly higher levels^23^. In addition, MPCs expression of BDNF and other neurotrophic factors can be potentiated by culturing these cells in a neurotrophic induction medium^22,23^. However, the neurotrophic induction protocol requires culturing the cells for 10 to 14 days in two different media, which may be impractical from a clinical point of view. Here, we show that modulation of a single factor, CTDSP1, can achieve the same effect in a shorter period of time. Importantly, we show that the increased expression of BDNF after CTDSP1 knockdown is associated to an increase in the secretion of BDNF from MPCs. This increase is sustained for at least one week after the transfection of siRNA. This is an important finding because the release of neurotrophic factors is fundamental for support cells at the site of injury to promote axon regeneration.

Another important finding of this study is that CTDSP1 knockdown promotes neurite regeneration. A caveat of this experiment is that we did not transect axons, but measured neurite outgrowth in culture after dissection and dissociation of DRG neurons. However, it can be argued that dissection mimics axotomy as the whole nerve is severed from the neuronal cell bodies in the DRG during the procedure. Accordingly, axotomy of DRG neurons during dissection and dissociation of the neurons has been shown to induce upregulation of regeneration-associated genes and activation of injury-activated transcription factors^28,29^. In addition, it has been shown that dissociating neurons from dorsal root ganglia activates a pro-regenerative program similarly to that occurring after a nerve injury^25^. The molecular and functional features of the pro-regenerative state have been reported to be active within the first 24 h of culture^25^. Therefore, neurite growth after DRG neuron dissociation can be considered a robust model of axonal regeneration.

Our findings also show an increase of CTDSP1 and decrease in BDNF mRNA in injured muscle tissue. The site of injury contains a heterogeneous population of cells, and the increase in CTDSP1 after injury may therefore reflect the change in the type of cells that are present at the site of injury, such as the presence of injury-induced cells, like MPCs, which may express higher levels of CTDSP1 and lower levels of BDNF than muscle cells. Nevertheless, insufficient levels of BDNF and other neurotrophic factors at the site of injury are partly responsible for inhibition of axonal regeneration^5,30^. Importantly, our study suggests that it may be possible to increase the secretion of BDNF and other neurotrophic factors at the site of injury by modulating CTDSP1 activity.

Although we have not directly investigated the mechanism through which CTDSP1 promotes neurotrophic factor expression and neurite regeneration, it is reasonable to hypothesize that the effects of CTDSP1 knockdown are a direct consequence of REST degradation due to lack of CTDSP1-mediated dephosphorylation of serines 861/864^12^. Several lines of evidence support this hypothesis. It has been shown that CTDSP1 is recruited to the REST complex and its levels vary in parallel to REST levels during differentiation. For instance, similarly to REST, expression of CTDSP1 decreases as stem cells differentiate into neurons^31^, while knockdown of CTDSP1 in neuronal progenitor cells accelerates neuronal differentiation^32^. Although CTDSP1 was initially identified as a phosphatase for the C-terminal domain of RNA polymerase II^33^, it has been shown to silence neuronal genes specifically, without effect on general transcription^31,34^. However, we cannot exclude an alternative action of CTDSP1 on modulation of neurotrophic factors levels and nerve regeneration.

Our results show a small, non-statistically significant difference between REST and CTDSP1 knockdown in promoting the expression of BDNF and NGF. This difference may be due to the activity of structural and functional paralogs of CTDSP1, namely CTDSP2 and CTDSPL, which are not affected by CTDSP1 siRNA^33,35,36^.

In summary, the decreased expression, in both neurons and supporting cells, of the genes required to sustain neuronal survival and promote axonal growth and synaptic plasticity, happens concurrently to an overexpression of REST. Preventing REST-mediated repression of transcription should promote nerve regeneration through the expression of neural genes and neurotrophic factors that have been shown to facilitate axonal regrowth after injury. Our study suggests that inhibition of CTDSP1 activity allows the simultaneous modulation of the REST pathway in both neurons and the support cells located at the site of injury. Our findings support the development of an epigenetic reprogramming strategy to promote end-organ reinnervation and functional recovery after PNI through inhibition of CTDSP1. This is a novel approach that focuses on removing the epigenetic repression of genes required for successful regeneration, end organ re-innervation and synapsis formation.

## Methods

### Human muscle tissue

Traumatized human muscle tissue was collected during surgical debridement following orthopaedic injury to the extremities at Walter Reed National Military Medical Center. The tissue was collected at the margin of devitalized and healthy appearing tissue and would otherwise be discarded as surgical waste. Control non-traumatized tissue was obtained during harvest of the tendon for anterior cruciate ligament surgery. The Walter Reed National Military Medical Center Institutional Review Board waived the need for consent. No identifiers were included with the specimens. Surgeons performing the debridement surgeries were not associated with the study. All procedures were performed in accordance with relevant guidelines and regulations.

### Mesenchymal progenitor cell (MPC) harvesting and culture

MPCs were isolated from traumatized human muscle tissue collected as described above. MPCs were harvested as previously described^37,38^. Briefly, muscle tissue was incubated for 2 h at 37 °C in DMEM with 0.5 mg/ml collagenase 2 (Worthington Biosciences). To obtain pure MPC cultures, the dissociated cells were then pelleted by centrifugation and plated in a T150 tissue culture flask in DMEM (GIBCO) containing 10% v/v fetal bovine serum (FBS; Invitrogen) and 5 U/ml penicillin, streptomycin and fungizone (PSF; Invitrogen). After 2 h, non-adherent cells were removed by washing with phosphate-buffered saline (PBS; Invitrogen) and the adherent cells (MPCs) were cultured in growth medium (DMEM supplemented with 10% v/v FBS and 1 U/ml PSF).

### HEK-293 cell culture

HEK-293 cells (ATCC-CRL-1573) were cultured in growth medium (DMEM supplemented with 10% v/v FBS and 1 U/ml PSF).

### RNA isolation and RT-qPCR

Cells or tissue were lysed in QIAzol (Qiagen) and total RNA was extracted using RNeasy Midi Kit (Qiagen) according to manufacturer’s instructions. In the case of muscle tissue, about 50 mg of muscle tissue were placed in a microcentrifuge tube containing Bullet Blender beads (NextAdvance) and homogenized in a Bullet Blender homogenizer using a volume of Qiazol 10 × the weight of the tissue. Purified RNA was quantified with NanoDrop 2000 (ThermoFisher), and reverse transcription was run using High Capacity cDNA Reverse Transcription Kit (Applied Biosystems) and 2 ng/ml RNA per reaction. For qRT-PCR, 5 ml of cDNA (corresponding to 10 ng of RNA) were mixed with to 10 ml SsoAdvanced Universal SYBR Green Supermix (BioRad) and 1 ml of each primer (final primer concentration 500 nM each). Reactions were run in triplicates in a QuantStudio 7 Flex Real-Time PCR system (Applied Biosystems). Amplification data were analyzed using the comparative cycle threshold (ΔΔCt) method and β-actin as calibrator. The primers used were as follows: human β-actin forward 5'-AGAGCTACGAGCTGCCTGAC-3’, human β-actin reverse 5'-GGATGCCACAGGACTCCA-3’; human REST forward TCAGCATGTTAGAACTCATACAGGA, human REST reverse TCTTCTGAGAACTTGAGTAAGGACA; human CTDSP1 forward CGCCATCCCTAAGCAGAC, human CTDSP1 reverse CCACAGGGATGATGAAGTCC; human BDNF forward 5’TATTAGTGAGTGGGTAACGGCG3’, human BDNF reverse 5’GAAGTATTGCTTCAGTTGGCCTT3’; human NGF forward 5’TATCCTGGCCACACTGAGGT3’, human NGF reverse 5’TCCTGCAGGGACATTGCTC3’; rat β-actin forward AGAGCTATGAGCTGCCTGAC, rat β-actin reverse GGATGCCACAGGACTCCA; rat REST forward CTGGTGGAACTCAGGGTCC, rat REST reverse GGGTCACTTCGTGCTGATTAGAGG; rat CTDSP1 forward TTACTCAGATCAGCAAGGAGGAGG, rat CTDSP1 reverse CTGCTGACTTCTGGTCACCTTTG; rat BDNF forward GGACCAGGAGCGTGACAAC, rat BDNF reverse CTGGTGGAACTCAGGGTCC .

### Western blot

Cells or tissue were lysed in RIPA buffer with 1:100 Halt protease inhibitor cocktail (both from Thermo Fisher Scientific). Total protein was quantified with bicinchoninic acid (BCA) protein assay (Pierce Biotechnology) to equalize the protein loaded among samples. For SDS-PAGE, samples were denatured and loaded on a NuPAGE 4–12% Bis-Tri gel in MOPS (Invitrogen). Protein was transferred to nitrocellulose membranes using Power Blotter System (Invitrogen). Blots were blocked in 5% bovine serum albumin (BSA) for at least 1 h and incubated with the primary antibody diluted 1:1000 in TBS 0.1% Tween (TBS-T) at 4 °C O/N. After washing 3 × in TBS-T blots were incubated with the HRP-linked secondary antibody diluted 1:10,000 in TBS-T, for at 1 h at RT. Blots were then washed 3 × in TBS-T and the signal developed using Immobilon Western (Millipore). Images were taken with Bio-Rad Chemidoc imager (BioRad). For all experiments, GAPDH was used as loading control. The following antibodies were used: GAPDH (Cell Signaling Technology), REST (Millipore), CTDSP1 (Invitrogen).

### Enzyme-linked immunosorbent assay (ELISA)

Supernatant was collected from MPC cultures 1 and 8 days after siRNA transfection. BDNF concentration was quantified with the Human/Mouse BDNF DuoSet ELISA kit (R&D Systems), using an Infinite M200 Pro microplate reader (Tecan).

### RNA knockdown

Cells were transfected with REST or CTDSP1-targeting siRNA or a control scramble siRNA (Silencer Negative Control No. 3 siRNA) (all from Invitrogen) using RNAimax (Invitrogen) according to the manufacturer’s instructions. Briefly, siRNAs and RNAimax were diluted in Opti-MEM (Thermo Fisher Scientific) and added directly to the culture medium. For knockdown experiments in HEK-293 cells, fluorescently labeled siRNAs (Invitrogen) were used and cells were sorted by fluorescence activated cell sorting (FACS) twenty-four hours after transfection to select cells that had incorporated siRNAs. The siRNA sequences were as follows: human *REST*: 5’GGCAAGAGCUCGAAGACCA3’; *CTDSP1*: 5’GGACUCAGACAAGAUCUGC3’.

### Fluorescence activated cells sorting (FACS)

For FACS, cells transfected with fluorescently-labeled siRNA were resuspended in 2% serum in PBS at a concentration of 10^6^ cells/ml, and filtered through FACS tubes with 35 mm strainer cap (Falcon). Cells were sorted with a BD FACSAria Fusion Cell Sorter (BD Biosciences). Cells expressing the 30% highest fluorescence intensity were collected for culturing as described above.

### Surgical procedure

For sciatic nerve injury, Sprague–Dawley rats (200-225 g) were anesthetized by intra-peritoneal injection of a mixture of ketamine (90 mg/kg) and xylazine (10 mg/kg). After shaving the surgical site and cleansing the skin with betadine, a 3 cm incision was made on the lateral left thigh with a surgical scalpel. The cranial and caudal parts of the biceps femoris muscle was bluntly dissected and separated with a self-retaining retractor to expose the sciatic nerve and its three terminal branches: the sural, common peroneal, and tibial nerves. The sciatic nerve was transected above the terminal branches and an immediate microsurgical epineural repair was performed using two to three 9-0 nylon sutures. The muscle and skin were closed in two layers with Monocryl Plus 6-0 sutures and Ethilon 4-0 sutures, respectively to avoid any opening of the wound by biting. The sham surgery group had the sciatic nerve exposed, but left intact.

All animal procedures were performed under an approved appropriate protocol by the Institutional Animal Care and Use Committee at the Uniformed Services University of Health Sciences. Animal experiments were performed in compliance with the “Animal Research: Reporting of In Vivo Experiments” (ARRIVE) guidelines (https://arriveguidelines.org/).

### DRG neuron culture

For collection of DRGs, Sprague–Dawley rats (200–225 g) were euthanized with an overdose of ketamine/xylazine followed by decapitation, under an approved protocol by the Institutional Animal Care and Use Committee at the Uniformed Services University of Health Sciences. DRGs were dissected from adult rats and kept in L-15 during dissection. Dissected DRGs were then digested in 1% collagenase on a shaker in a cell culture incubator (37 C, 5% CO_2_) for 1 h. Collagenase was then replaced with 0.25% trypsin for 30 min. Trypsin activity was blocked by adding DMEM supplemented with 10% v/v fetal bovine serum (FBS; Invitrogen), and the DRGs were centrifuged at 300 × g for 10 min. DRGs were resuspended in 1 ml DMEM 10% FBS and triturated by repeated pipetting. The mixture was then filtered through a 100 mm cell strainer to discard fibrous and undigested tissue. The dissociated cells were added to the top of 4 ml of 15% BSA in DMEM/F12 and centrifuged at 300 × g for 10 min. The pellet containing the DRG neurons was resuspended in Neurobasal-A medium (Gibco) supplemented with B27 (Gibco), 50 ng/ml NGF (Sigma-Aldrich) and penicillin streptomycin and fungizone (Invitrogen) and cells were plated on dishes pre-coated with PDL and laminin. To prevent proliferation of non-neuronal cells, 25 mM 5-fluoro-2′-deoxyuridine (Sigma-Aldrich) was added to the medium on the day of plating.

### Neurite length measurement

Neurites growing from cultured DRG neurons were imaged by phase-contrast and interference light microscopy using an Axio Observer Z1 with Apotome optical sectioning device (Carl Zeiss). The longest neurite was traced and its length measured with Simple Neurite Tracer (FIJI, ImageJ).

### Statistical analysis

All statistical analyses were performed with GraphPad Prism 8 (GraphPad Software, Inc.). In case of non-normal distributions, Mann-Whitney or Kruskal-Wallis test followed by Dunn’s multiple comparisons test were used. The statistical values are reported as mean ± standard deviation (SD) or mean ± standard error of the mean (SEM), as described in the figure legend. Statistical significance was set at α = 0.05 for all experiments. Data are representative of at least three independent experiments. The statistical test performed, the number of replicates and the error bars shown for each experiment are described in the figure legend.

### Ethics approval and consent to participate

The Walter Reed National Military Medical Center Institutional Review Board approved the human tissue procurement protocol and waived the need for consent. All animal procedures were performed under an approved appropriate protocol by the Institutional Animal Care and Use Committee at the Uniformed Services University of Health Sciences.

## Supplementary Information

Supplementary Information.

## Data Availability

All data generated or analyzed during this study are included in the published article.
