# Supplementary material for: C-terminal domain small phosphatase 1 (CTDSP1) regulates growth factor expression and axonal regeneration in peripheral nerve tissue
Source: Sci Rep. 2021 Jul 14;11:14462. doi: 10.1038/s41598-021-92822-8 (PMC8280205; doi:10.1038/s41598-021-92822-8)
Supplement: Supplementary file 1 — Supplementary Information. [file 41598_2021_92822_MOESM1_ESM.pdf]

# C-terminal domain small phosphatase 1 (CTDSP1) regulates growth factor expression and axonal regeneration in peripheral nerve tissue


## Authors:

Noreen M. Gervasi[1,2,3], Alexander Dimtchev[1,2], Desraj M. Clark[1,4], Marvin Dingle[1,4], Alexander V. Pisarchik[3*], Leon J. Nesti[1,4*]

## Affiliations:

[1] Laboratory of Clinical and Experimental Orthopaedics, Department of Surgery, Uniformed Services University of Health Sciences, 4801 Rockville Pike, Bethesda, MD 20889, USA.

[2] Henry M. Jackson Foundation for the Advancement of Military Medicine, 6720A Rockledge Drive, Bethesda, MD 20817, USA.

[3] Alcamena Stem Cell Therapeutics, 1450 South Rolling Road, Suite 4.069 Halethorpe, MD 21227, USA.

[4] Department of Orthopaedics and Rehabilitation, Walter Reed National Military Medical Center, 8901 Rockville Pike, Bethesda, MD 20889, USA.

*Correspondence to: leonnesti@gmail.com; leon.nesti@usuhs.edu; leon.j.nesti.mil@mail.mil; a.pisarchik@alcastem.com

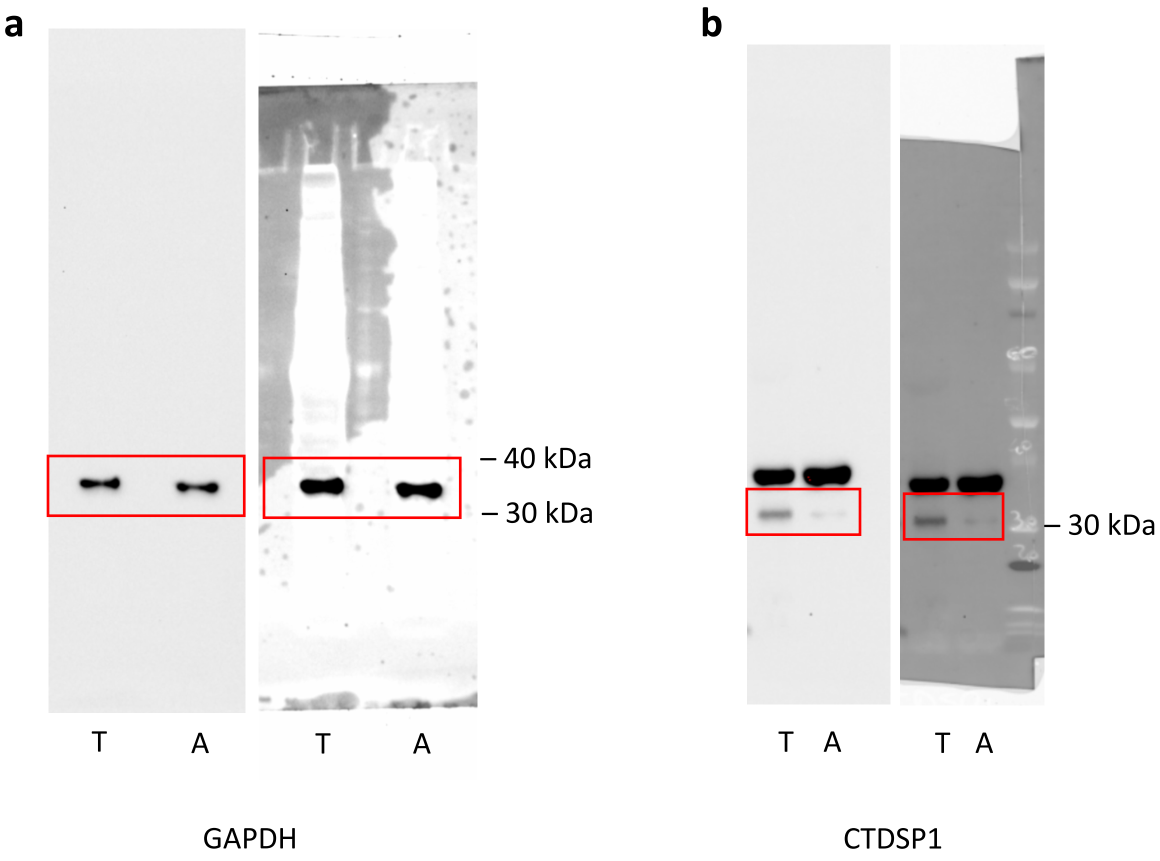


**Supplementary Figure S1.** Figure 2a full-length blots. Multiple exposures are shown. **a** GAPDH blot. For the final figure, the blot was cropped to eliminate the empty lane in between the bands. **b** CTDSP1 blot. The blot on the right was superimposed with an image of the protein ladder to show the molecular weight of the bands. For the final figure, the blot was flipped horizontally and cropped to show the specific CTDSP1 band at about 30 kDa. T = traumatic, A = atraumatic.

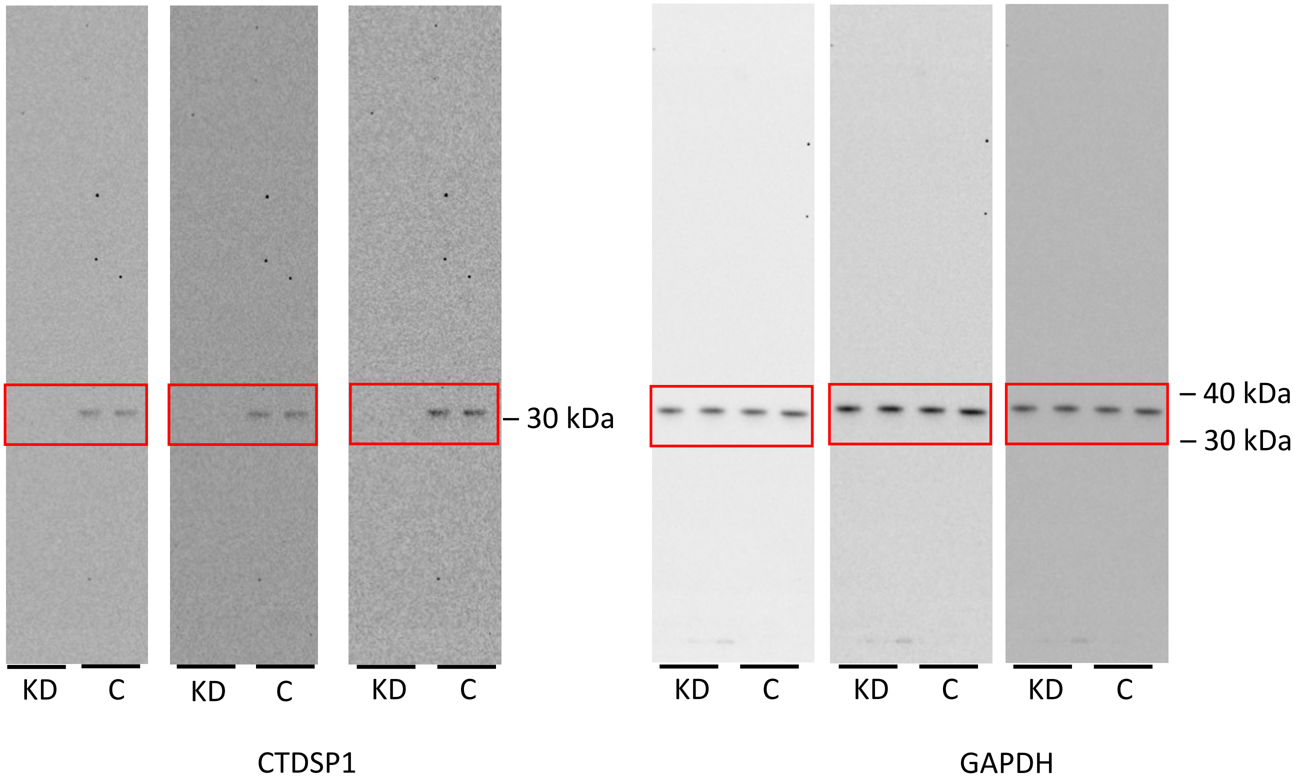


**Supplementary Figure S2.** Figure 3a full-length blots. Multiple exposures for CTDSP1 blot are shown. For the final figure, blots were flipped horizontally. KD: CTDSP1 knockdown, C: control

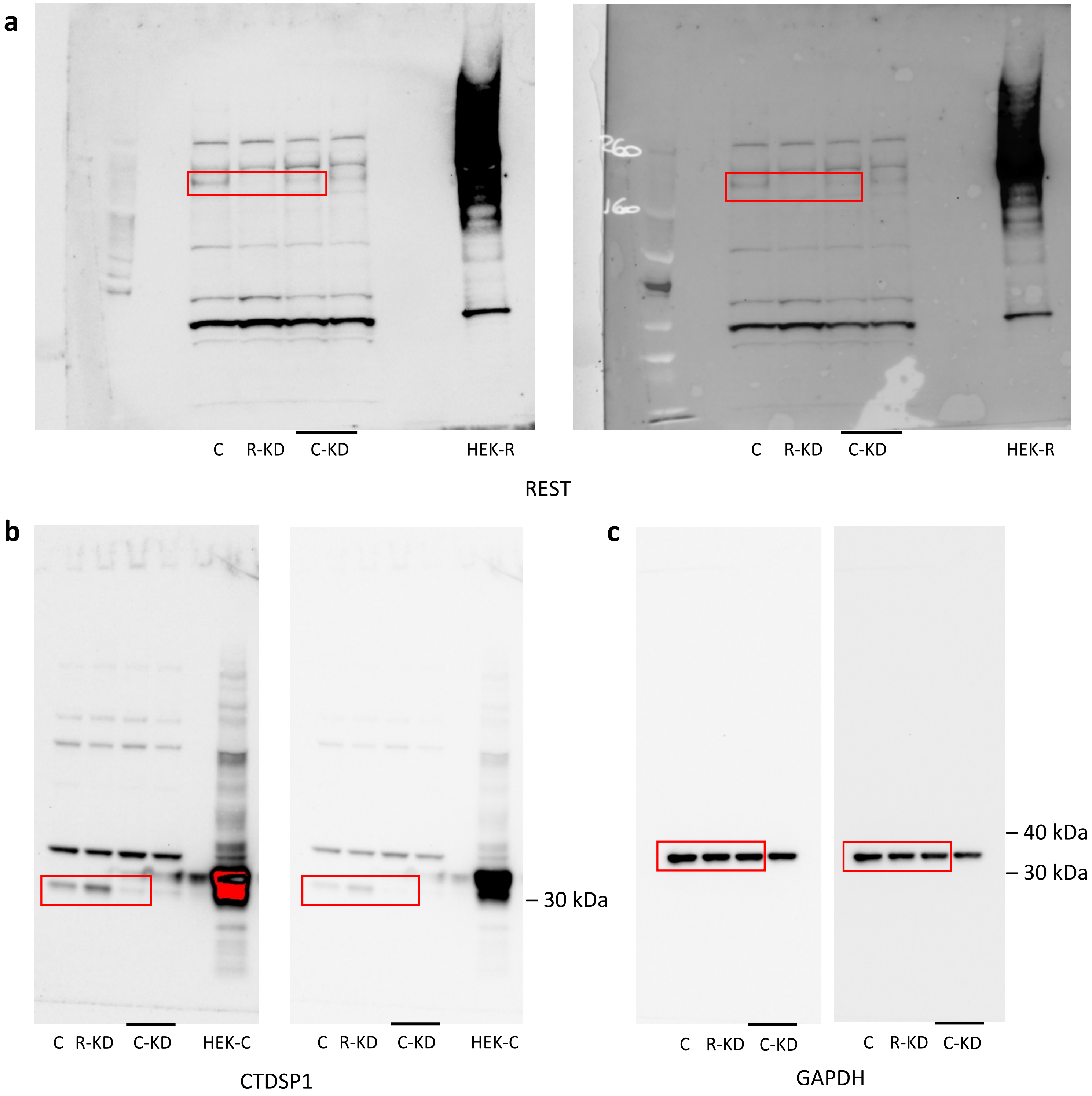


**Supplementary Figure S3.** Figure 3c full-length blots. Multiple exposures are shown for REST (**a**), CTDSP1 (**b**) and GAPDH (**c**). For REST and CTDSP1, band specificity was verified with lysate from HEK-293 cells transfected with a plasmid encoding REST and CTDSP1, respectively. REST blot: Image on the right was superimposed to an image of the protein ladder. L = protein ladder C = control, R-KD= REST knockdown, C-KD = CTDSP1 knockdown, HEK-R: HEK-293 cells over-expressing REST HEK-C: HEK-293 cells over-expressing CTDSP1.
